# Selections and Abstracts

**Published:** 1899-07

**Authors:** 


### *SELECTIONS AND ABSTRACTS.*

---

### MOSQUITOES AND MALARIA.

The history of the theory that there is a connection between mosquitoes and malaria is interesting, and would be curious did not the annals of science afford many parallels. The theory appears first of all to have been thrown out by Crawford, who published a paper on the "Mosquital Origin of Malarial Disease" in the *Baltimore Observer* as long ago as 1807. Nobody seems to have revived it seriously until 1883, when King published in the *Popular Science Monthly* a paper entitled "Insects and Disease, Mosquitoes and Malaria." Still, it made little way, or, rather, lay dormant, until Manson, by a really brilliant piece of inductive reasoning showed, in his Gulstonian Lectures of 1896, that the natural history of the disease and the life history of the mosquito afford the grounds for looking upon the hypothesis as reasonable. He also indicated the lines upon which research to settle the question should be conducted. The admirable work which Major Ronald Ross of the Indian medical service has since done on these lines is well known to our readers, for his papers and reports have been for the most part published in our columns. Dr. Manson gave an account of the results of the research down to last July in an address in the section of topical medicine at Edinburgh, which was published in the *British Medical Journal* of September 24, 1898. * * *

M. Laveran, the discoverer of the hematozoon of malaria, who has for some time shown that he is impressed by the weight of the evidence in favor of the mosquito theory, brought the subject before the Académie de Medicine on January 31, when he paid a warm tribute to the excellent work done by Ross. His commmunication took the form of a report on Ross's paper, and in introducing the subject he said that the hypothesis that the hematozoon of malaria might probably exist outside the human body as a parasite of mosquitoes was at first received with much skepticism, but had gradually made its way, and was now accepted by most writers on malaria. Manson in England, Koch in Germany, and Bignami and Grassi in Italy, had all published very interesting obversations on the point; but it is, he said, "the patient researches of Ronald Ross which have put beyond the range of doubt the part that mosquitoes play in the complicated evolution of the hematozoon of malaria and of the endoglobular hematozoon of birds, which is very nearly allied to malaria."

M. Laveran said that he had been for some years in correspondence with Ross, from whom he had received histological specimens showing the transformation of the hematozoon in mosquitoes. "I can, therefore," he said "bear witness to the admirable perseverance which our distinguished confrère has shown in the study of this difficult question, and to the exactness of his observation." M. Laveran then traced the course of Ross's researches since 1895, and said that in their course he had discovered a series of very interesting facts. Ross had been the first to observe the transformation of the hematozoon of malaria in certain mosquitoes; he had been the first to see, and to describe with precision, the transformations of the proteosoma of birds in the digestive canal and body cavity of mosquitoes. Finally, he had succeeded in infecting healthy birds by causing them to be bitten by mosquitoes which had fed on diseased birds.

M. Laveran then pointed out that further investigations were particularly required with regard to the following points: 1. The manner in which the proteosoma introduced into the stomach of mosquitoes becomes transformed into the pigmented spherical bodies which are found after thirty hours in the substance of the stomach wall. 2. The mode of formation and the nature of the black spores and the part which they play. 3. The mode of formation and the structure of the germinal threads. 4. The determination of the species of mosquitoes which act as hosts. 5. The verification of the theory that mosquitoes play the same part in transmitting the hematozoon to man as Ross has shown they play in carrying a similar parasite to birds. M. Laveran observed that Bastianelli, Bignami, and Grassi had followed the development of the crescentic bodies in the intestines of mosquitoes which had sucked the blood of patients suffering from Italian summer-autumn fever, and that the changes observed were precisely those described by Ross in the case of the proteosoma. Finally, Bignami recently succeeded in giving an individual malaria by exposing him for several consecutive nights to mosquitoes obtained from a malarious district.

It was not, M. Laveran continued, surprising, having regard to the great difficulty of these researches, that an isolated observer working as Ross did, often under defective conditions, had not succeeded in solving all these problems; but, thanks to his discoveries, it might now be hoped that it would be easier to elucidate the remaining questions, since investigators had been put on the right road, and on all sides were beginning to verify and complete Ross's researches. In conclusion, M. Laveran sincerely congratulated Major Ross on the important contribution that he had made to the history of the evolution of the parasite of malaria outside the human body, and the Academy adopted a special vote of thanks to him for the interesting communication which he had addressed to it on the subject.

Professor Koch also has recently issued a report on the results reached by the German commission which spent last August and September in Italy in the study of malarial disease. Professor Koch was the head of the commission, and the other members were Professors Pfeiffer and Kossel.* The subjects to which the commission specially directed their attention were: 1. The relationship of the different forms of malaria grouped together under the name of febri estivo-autumnali; (2) the connection between

* Deutsche Medicinische Wochenschrift, February 2, 1899.

Italian and tropical malaria; and (3) the etiology of malaria, especially in reference to its transmission by blood-sucking insects.

With regard to the first point, Professor Koch reports that the commission came to the conclusion that the febri estivo-autumnali, though they could be divided clinically into several varieties, were all caused by a single form of parasite. Cases newly developed were all of the same tertian type, and it was only when their course was interfered with by the action of quinine or of a naturally acquired immunity that they became quotidian and finally irregular.

With regard to the second point, the opinion is expressed that there is no essential difference between the malaria of Italy and that of the tropics. Distinctions reported by Italian observers were shown by improved methods of research to be artificial. This Professor Koch considers to be a great step in our knowledge of malaria.

Light was also thrown on the third point. It had been assumed by some that the so-called semilunar forms and the flagellated bodies derived from them were degenerated forms of the malarial parasite, mainly on the ground that they did not take on a chromatin stain—a sure indication that the reproductive powers of an organism have been lost. Owing to improvements in Romanowsky's staining method, however, it was discovered, first, that there were chromatin bodies in the semilunar forms; and, secondly, that the so-called flagella contained chromatin, and were really analogous to spermatozoa.

Koch was able to recognize in Italy the parasite (proteosoma) in birds, the development of which Ronald Ross has described. He was able to confirm Ross's statement that after the blood infected with proteosoma has reached the stomach of the mosquito, a number of coccidia-like bodies make their appearance, and that within these spores develop, some of which ultimately reach the poison-salivary glands, whence they are injected into the victim's skin.

Professor Koch and his colleagues were thus able completely to confirm Ross's observations, and they express the opinion, founded on the close resemblance of the malarial parasite to the proteosoma, that it is probable that the two have a similar life-history. Ross's work is therefore, they conclude, of the greatest value as a guide to future workers in the field of human malaria.

The report points out that the city of Rome itself, though it lies in the midst of a wide expanse of malarious country, is nevertheless (at all events as far as the central part is concerned) free from malaria. This immunity cannot be due to its water or food supply, for its water, vegetables, and fruit come from malarious districts. In those parts of Rome, however, in which large gardens provide a habitat for mosquitoes, there malaria is found.

Koch mentions incidentally that certain cases of malaria in which quinine—from a fear of blackwater fever—was contraindicated, did very well on methylene blue. Some of the cattle of the Campagna were examined, and it was found that those of them which suffered from Texas fever were infested with the same species of tick which produces Texas fever in East Africa and in Texas itself.

The confirmation of Ross's work by an observer so experienced as Koch, and the high praise accorded to it by Laveran, will, we venture to expect, encourage the Indian government to afford still further opportunities and facilities to this distinguished member of the Indian medical service.—*British Medical Journal.*

---

### DON'TS FOR THE TREATMENT OF PNEUMONIA.

Dr. Thomas J. Mays, in the *Polyclinic,* gives the following list of "Don'ts" in the treatment of pneumonia:

Don't believe that acute pneumonia is a self-limited disease and will get along as well without treatment as with it.

Don't hug the delusion that fever in any degree is a benefit to the patient.

Don't fancy that you can always tell croupous from catarrhal pneumonia.

Don't allow pain in the abdomen to draw your attention away from the chest. Frequently the beginning of pneumonia is accompanied by severe pain in the right groin, which may lead one to suspect the onset of typhoid fever.

Don't direct your treatment more towards the heart than towards the lungs.

Don't fail to recognize the great influence of the brain and nervous system.

Don't lose sight of the serious indication of rapid and laborious breathing.

Don't be afraid to apply ice to the chest in rubber bags. It will do no harm.

Don't fail of applying as many bags as are necessary to cover the area of inflammation.

Don't think that you can get as good results from a tub bath or from cold general sponging, as you can from the local application of ice.

Don't become alarmed when the ice produces a sudden drop in the temperature and think the patient is going into collapse.

Don't fail to retain the ice so long as fever is present and resolution has not taken place.

Don't omit to apply one or two ice bags to the head.

Don't overlook the beneficial influence of strychnine in combating pneumonia. Administer 1-20 of a grain by the mouth every three or four, and besides give the same dose hypodermically once or twice a day, until the system becomes irritable.

Don't omit the hypodermic injection of $\frac{1}{4}$ of grain of morphia once or twice a day to secure rest and sleep.

Don't fail to administer oxygen by inhalation more or less constantly if the patient is cyanotic or short of breath.

Don't fail to bleed if cyanosis and dyspnea are not relieved by oxygen inhalation.

Don't lose sight of the great value of tincture of capsicum in relieving great nervous depression, delirium, dry, black-coated tongue, picking at the bed-clothes, etc. Give from a half to one teaspoonful doses in water every two or three hours or oftener, in alcoholic pneumonia.

Don't fail to give sodium salicylate, ammonium acetate, potassium acetate, and potassium citrate, three grains of each, in a dessertspoonful of peppermint water, every three or four hours, if there is the least evidence of a rheumatic complication.

Don't overlook the important action of quinine in this disease.

Don't fail to support the patient with an abundance of nourishing food, such as milk, freshly expressed beef-juice, etc.

---

### THE TIME-ELEMENT IN THERAPEUTICS.

A very important feature in the proper application of remedies has been overlooked by many authors (Merck's Archives), and is seldom much considered by practitioners. We refer to the time which elapses between the administration of a remedy and the beginning of its action, also the duration of such effect. In the relief of many conditions, and especially in the use of combined drugs, this point is a very important consideration. In the use of digitalis it is seldom taken into consideration that it does not reach the height of its action for from four to six hours after administration, and that the effect is often prolonged for a longer period. The administration of this drug, then, every three hours would hardly be justified. In several instances where it is the custom to prescribe remedies which have to a certain extent neutralizing or antagonistic effects it is important to know these facts, otherwise the desired result may not be accomplished. For instance, it is quite a common practice to combine with digitalis remedies which counteract its effect upon the arterioles as does nitroglycerin, but how often is the difference in the time of their action considered? Digitalis acts very slowly, and its action is prolonged, while nitroglycerin shows its action in a few seconds, and this passes off usually in two or three hours. These drugs are frequently prescribed in one mixture, often to be given every four hours. In such a course it will readily be seen that at the latter part of the interval the patient has the acquired effect of two doses of digitalis, while the effect of the nitro-glycerin has passed off. In cases where an immediate stimulation is indicated it is important to know how long we must wait for the desired effect. Nitro-glycerin is immediate, brandy by the stomach or hypodermically is rapidly diffused, and is more rapid than strychnine or many of the other commonly-used stimulants. Digitalis requires some time for the development of its stimulant action, even when given hypodermically.

Few of the works now published upon materia medica and therapeutics give the effect of different drugs. Many preparations manufactured by the pharmacist, both in the form of elixirs and tablets, show a lack of consideration of this very essential feature of scientific therapeutics. A general review of the remedies now in use with special reference to time as an element in the physiological action would fill an important place in the armamentarium of the practitioner.

---

### RETINAL AND LABYRINTHINE HEMORRHAGES IN A CASE OF SPLENIC LEUCEMIA.

James Finlayson (*British Medical Journal*) reports the case of a married woman, aged twenty-nine years, suffering from splenic leucemia, who contracted a "cold" which was followed by a suddenly developed ringing sound in the ears, giddiness, and nausea, accompanied by a bloodshot appearance of the left eye. There was a decided conjunctival ecchymosis, and an examination of the fundus showed the presence of hemorrhages. The ears presented no evidences of anything abnormal in the external and middle ear, and the Eustachian tubes were easily inflated, though without improvement in the hearing. The suddenness of the onset, with the symptoms of giddiness, etc., led to a diagnosis of hemorrhagic exudation into the cavities of the labyrinth. Post-mortem sections of the retina showed the hemorrhages noted during life, with a striking abundance of leucocytes in the blood-vessels. Examination of the internal ear revealed hemorrhages in the vestibule and in the first turn of the cochlea.

---

### PROPHYLACTIC GARGLE IN SCARLATINA.

The frequency of otitis media as a complication of scarlatina is said to be reduced to a minimum if, several times a day, the tonsils and pharyngeal vault be gently brushed with absorbent cotton wet with the following solution (Louisville *Med. Mon.*):

℞ Beta-naphthol .................................. dr. 1
Camphoræ ....................................
Glycerini ...................................... aa dr. 4

M. Sig.: For application to throat.

### "MEDICAL JOURNALISM."

Under the above title the *Boston Medical and Surgical Journal* prints in its editorial columns, July 22, 1898, an article that we need make no apology for reproducing in full. It contains much of interest to every medical editor and publisher, and every contributor to the medical press ought to read it carefully and ponder well its dignified sentiments and adopt its high moral tone.

Within the past few years the increase in number of medical weekly and monthly publications has been so enormous that all contributors, however unworthy, are easily accommodated, if not in one direction then in another. Everybody who styles himself "M.D." now finds abundant opportunity to air his views or solicit advice in a perplexing case, and the result is a species of medical journalism as variegated and heterogeneous as the "Purple Cow" or "Lavender Pig" exemplars of general literature (?) that are constantly appearing upon the news-stands.

A critical examination of the contents of certain specimens of the class of medical journals to which we refer reveals on the part of their contributors an ignorance of the most commonplace facts of medical science that is simply deplorable, and a sublime disregard for rules of syntax that is none the less deplorable, although infinitely more amusing. The conclusion to which the examiner inevitably comes is that the medical press of this country is in sad need of a censorship.

Dialect stories cannot compare, for bizarre spelling and phraseology, with the communications of certain medical gentlemen in distress over patients with "a touch of the consumption" or a complex of symptoms designated (shades of Charcot!) "a spell of the hysterics." From the intricacies of construction which characterize these writings, the Queen's English undergoes every variety of contortion until, finally, the very acme of elegant simplicity of diction is encountered in journals of the "correspondence" type. In these we find "Doc" So-and-so communicating his perplexities to "Doc" Somebody-else or bestowing upon him an infallible remedy for piles in exchange for a prompt and safe emmenagogue. All these little amenities are characterized by a style of diction that rivals the masterpieces of the "Ready Letter-writer" and demonstrate, if they do no more, that the lacteals of human kindness are never withered in the medical breast.

This is the class of writers about whom Dr. Holmes* says: "They go for weakness whenever they see it with stimulants and strengtheners, and they go for overaction, heat and high pulse and the rest, with cooling and reducing remedies." But their contributions can be of no positive value to scientific medical literature for the reason that they aid in no way toward a better understanding of disease processes. The movement which has set such a class of writings on foot is retrograde in that it tends to foster empirical methods of prosecuting a calling which now, more than at any other period of its existence, demands that its true representatives shall be scientists in the fullest sense of the word and not mere dispensers of powder and pellet.

The dignified attitude which medicine is beginning to assume among the sciences can never be maintained unless the writings which record its progress are of a quality to compare with those which mark the advances in the sister sciences.

We have gained so much of exact knowledge through pathological and experimental research, that future contributions to current medical literature should be stripped almost entirely naked of theory and speculation, those barriers to scientific progress. To achieve this the medical writer must needs be an investigator, at least to the extent of finding out how far such views as he is about to put forth are substantiated by the facts of pathology. The neglect of such a preliminary step is to-day a tacit avowal of both laziness and ignorance, since our library facilities for such work are abundantly adequate. When the writer has before him all necessary material, he should aim, above all, at clearness and accuracy. His English should be terse, not oratorical and certainly not colloquial; it should be free from hybrid technical terms, especially those with Greek roots and Latin endings. Finally, his bibliography should be such as will bear verification, for there is nothing more maddening to the man who is searching the literature than to find reference after reference which resists all attempts at identification.

* The Poet at the Breakfast-table, Riverside Press edition, p. 121.

The only accurate way to refer is to refer in full, even to the page number of every quotation.

In conclusion, we wish to allude to two faults which are rife enough among contributors to medical journals to be considered annoying, to say the least. The first is the eternal dragging in of the *ego;* and the second, an inordinate fondness for the sensational. With respect to the first, we can only say that in our opinion no man's writings will ever lose anything of dignity or worth by being, as far as possible, impersonal. As a sample of the second fault we need only call the reader's attention to the earlier literature of the *x*-ray. If he will but compare that with what represents the thoughts of men who have probed its usefulness to the bottom, the moral lesson will be obvious.—*Buffalo Med. and Surg. Journal.*

---

### SLEEP IN TREATING DISEASE.

Wm. Evart, in an address delivered before the Harveian Society (*British Medical Journal*) on "Disease and its Treatment," has the following to say on the value of sleep: "Sleep has two offices, both fulfilled in the long sleep of the night, which it is our usual endeavor to secure for our patients, namely, that of favoring the slow changes of repair, and that of interrupting consciousness by uncoupling the chain of neurons, or by relaxing protoplasmic tension or tone. This relief of tension is, it would seem, the only office performed by the shorter spells of sleep, and therefore the two forms of sleep suggest two therapeutic objects. The night's sleep which comes without any drugs may need to be bettered, and in improving the quality of spontaneous sleep our help is often of value. It might also need to be prolonged.

"The systematic prolongation of sleep for the cure of disease is one of our opportunities hitherto little used. In chorea, sleep entirely subdues the muscular agitation, and this observation has led to the attempt to arrest the disease by prolonging sleep for considerable periods. A complication arises in connection with alimentation which in this disease, as in most other nervous troubles, is of primary importance. Partly for this reason, and because more than rest may be needed for a cure, the results hitherto reported have not sufficiently recommended the method.

"Prolonged narcosis has also been suggested in excessive wear and tear of the nervous system; and in various nervous affections, including the mental, its renewed trial, combined with suitable methods of feeding, might lead to encouraging results.

"Best suited, perhaps, to our every-day needs is a systematic resort to the shorter sleep. Like the light instalments of food which restore the lost function of appetite and digestion, short sleep in the day may be essential to the cure of nocturnal insomnia. Our growing wealth in hypnotics warrants a hope that a suitable agent may yet be found which in that direction would minister to the health of the invalid, and might command the luxury of sleep at any opportune time for the convenience of the worker.

"Body rest as a systematic therapeutic agent has long found its place in our treatment for patients whom weakness alone, in the absence of medical advice, would not have compelled to take to their bed. To that class belong the frail women in whom the debility of anemia, of dyspepsia, and of overfatigue develop symptoms often mistaken for hysteria. Rest in bed is their first need. In the treatment of chlorosis this is now recognized as the essential element for a rapid recovery. Its methodical employment forms an essential part of the Weir Mitchell plan. But its most striking instance is that of the open-air rest cure for phthisis, which within quite recent years has largely replaced at foreign sanatoria the previous method by muscular exercise."

---

### TREATMENT OF POSTERIOR URETHRITIS.

The St. Paul *Medical Journal* gives the following abstract of a paper on this subject by A. Ravoyli: The author treating of posterior urethritis states that the greatest advance in the treatment of gonorrhea has been achieved by the school of Breslau through the studies of Neisser. The principle is based upon the destruction of the gonococcus as rapidly as possible. The Janet method of irrigating is shown to have originated in the possibility of overcoming the contraction of the cut off muscle at the neck of the bladder. The fluid preferred is 1 to 5000, to 1-1000 permanganate of potassium solution. The irrigation should be so used as not to produce much distension of the urethra; this can be done by not closing the meatus completely until the fluid has begun to flow into the bladder. The author prefers in treating the posterior urethra a recurrent catheter of his own design by means of which the solution washes the posterior urethra thoroughly without filling the bladder. The irrigation is kept up for five or ten minutes with the patient in the standing position.

In subacute cases he follows the permanganate irrigation by injecting a 1 to 400, to 1-100 solution of protargol. In inveterate cases associated with infiltration of the submucous tissues he recommends the introduction of a fenestrated sound with a 25 per cent. salve of ichthyol.

In sixty cases treated after this method recovery took place in from two to four weeks. Complications did not occur. Relapses occur in 20 per cent. of cases, mostly caused by indiscretion. Balsams internally were condemned.

---

### DERMATITIS PRODUCED BY BORIC ACID AND BORAX.

R. B. Wild (*The Lancet* of January 7, 1899), in recording a case of intoxication from the use ot boracic acid, says: "The affected skin was of a bright red color and covered with profuse scales of a slightly greasy character. The patches of disease were irregular in shape, roughly symmetrical in distribution, and very extensive in area, leaving smaller patches of healthy skin on the trunk and the proximal parts of the limbs. The hands and the forearms and the feet and the legs below the knees were uniformly red, scaly, swollen, and they pitted on pressure, Desquamation on the palms and soles occurred in large flakes. The scalp was red and scaly; the hair had almost entirely disappeared from the head, and was very thin on the face and pubes. The face presented only a few scaly papules. Digestion was disturbed, the appetite was poor, and there was marked debility and anemia with loss of flesh."

### ACUTE BRONCHITIS.

Potter recommends the following:

℞ Antimonii et potass. tart....................gr. 2
Liquor ammon. acet....................oz. 4
Spr. etheris nitrosi....................oz. 1
Tinct. aconiti ....................dr. ½
Syr. simplicis ....................q. s. oz. 6

M. Sig. A teaspoonful every two or three hours in the first stage.

Bartholow has used this with good results:

℞ Tr. sanguin....................
Tr. lobeliæ ....................aa dr. 1
Vini ipecac ....................oz. 2
Syr. tolutani....................q. s. ad oz. 4

M. Sig.: A teaspoonful every three hours.

---

### DIARRHEA.

Dr. T. B. Greenley has used this prescription in diarrhea for several years, and finds it almost a specific, especially so in summer complaint of children.

℞ Paregoric....................oz. 2
Ext. witch-hazel....................oz. 1
Carbolic acid....................dr. 1
Fld. ext. kino....................dr. 2
Jamaica ginger....................dr. 2
Precipitated chalk....................oz. 1
Simple syrup to make....................oz. 8

Mix thoroughly and always shake bottle well before using. For an adult a teaspoonful, repeated at intervals of three hours, until desired effects result. Dose for children in proportion to age.

---

### PRURITUS ANI.

*Le Progrès Médical* attributes the appended formula to Penzoldt:

℞ Sodium hyposulphite....................gr. 4½

Carbolic acid ....................dr. 1¼

Glycerin....................dr. 5

Distilled water....................oz. 14

M. Sig.: For external use. Apply frequently upon saturated compresses.

### SYPHILIS.

The treatment of syphilis by the internal administration of mercurial ointment finds many followers in continental practice. It is stated that the metal is better absorbed in this form. L. Silberstein (*Therap. Monats.*) prescribes it in pills thus:

℞ Mercurial ointment with hydrous wool fat basis..gr. 4.5
Powdered liquorice root........................gr. 5
Glycerin........................................gtt. 5
Mucilage of acacia, q. s. to mass.

Divide into 60 pills; each pill contains 0.025 gramme of mercury.

---

### SIROP DE L' JESUS.

This calming syrup, employed in young children for the relief of insomnia, convulsions, etc., is said (*Bull. de Pharm. de Lyon*) to represent in each teaspoonful:

℞ Potass. brom..................................
Sodii brom......................................
Ammon. brom.....................................
Calcium brom....................................aa 0.05 cgm.
Syr. belladonna (Fr. Cod.)...................... 1 gm.
Syr. aurantii flor.............................. 5 gm.

Dose: One to four teaspoonfuls according to age.

---

### IRRITABLE BLADDER AFTER CONFINEMENT.

Dr. W. E. Fothergill finds the following prescription useful:

℞ Salol........................................
Tinct. hyoscyami...............................aa dr. 2
Infus. buchu ..................................q. s. ad oz. 6

M. Sig.: Tablespoonful three times a day.

---

### RICKETS—RACHITIS.

Powell recommends the following:

℞ Syr. ferri iodidi.............................dr. 1
Syr. zingiberis.................................oz. 1
Aq .............................................q. s. ad oz. 3

M. Sig.: Dose, one dram, t. i. d. for a child of two years.

### THE TREATMENT OF ASTHMA.

BY ROBERT C. KENNER, A.M., M.D., LOUISVILLE, KY.

There is perhaps no disease which is more distressing to the patient than asthma.

The paroxysms of dyspnea are very agonizing and often persist for a considerable length of time. The burden on the heart is very great when the attacks are frequently repeated, or continued for a long time.

It is entirely foreign to the purpose of this paper to discuss at length the etiology of asthma.

I hold with Biermer, that the true explanation of the symptoms is a "spasm of the muscular tissue of the small bronchii. The causation of this condition has both constitutional and exciting elements. The constitutional elements of causation comprise a neuropathic tendency, nerve lesions, epilepsy and other affections of the nervous system. According to the best observers, males have the disease more frequently than females, although my notes show about an equality in the number of males and females. Exciting causes comprise climatic influences and individual susceptibilities to certain odors. Some patients are at once seized with an attack of asthmatic dyspnea when they inhale the odor ot ipecac and other drugs. Others cannot bear to have certain animals, flowers or vegetables brought near them. All these are factors which may be said to be true of individuals, but which cannot be classified.

In about twenty per cent. of my cases I have found chronic bronchitis associated with asthma, and emphysema was present in about fifteen per cent.

The treatment of asthma should be considered under two heads: First, relief of dyspnea; second, systemic.

To carry out the first indication is a matter of great importance, since the burden on the heart during an attack or dyspnea is very great. To relieve these attacks surely and speedily must therefore be our first aim. To accomplish this purpose reference to the text-books will show that a great number of remedies have been employed. Bromide of potassium, spirits ether comp., belladonna and all of the sedatives, anodyne and anesthetic remedies have been used. Many physicians give chloroform by inhalation to relieve the dyspnea. Ipecac and lobelia were the remedies which the older practitioners depended upon to accomplish this purpose. But, with a better comprehension of the factors present in these cases, remedies such as ipecac, lobelia, chloroform and even the bromides, and in a word, remedies which add additional burden to the heart are losing favor.

To overcome the attacks of dyspnea I have employed only nitroglycerin, strychnine and morphine hypodermic tablets (Sharp & Dohme's). These tablets at once relieve the dyspnea, and do not act as a cardiac depressant as is the case with the anti-spasmodic, sedative or anesthetic drugs usually exhibited in this condition. Conversely, however, the strychnine in these tablets acts as a cardiac stimulant, and shortly after the hypodermic injection we can discern an improvement in the volume of the pulse. The action of these tablets is prompt and improvement of the symptoms is manifest in a very short time. Often entire breaking up of an attack of dyspnea is seen in twenty minutes after the hypodermic injection. The measures to be instituted to establish a cure of the asthma will of course depend to a large extent upon the conditions existing in the case under consideration.

When chronic bronchitis is present we will make little headway if we fail to give constructives and have the patient guard against the vicissitudes of the weather. In treating the disease also we shall have to administer remedies applicable in emphysema when that is present.

In my practice no single remedy has been found equal to the iodide potassium for extending the time between the attacks of dyspnea and affecting an entire cessation of these attacks. Of course this agent is conjoined in each case with such other drugs as are considered appropriate.

I give below the outline of several cases which have been treated on the lines laid down here, these being only a few of a great many tabulated cases:

Mrs. S. C., aged twenty-five, a delicate woman, eight months pregnant. She has had asthmatic attacks for the past week, which had increased in severity and frequency. This attack had persisted for more than an hour and the patient was almost exhausted from dyspnea. Her pulse was 120. I at once gave her a hypodermic injection of nitroglycerin, strychnine and morphine tablets, and this gave her relief in a short time, and her pulse increased in volume and was lessened in frequency. She was suffering with associated bronchitis and I gave her cod-liver oil made palatable after meals, and iodide of potassium in doses of five grains before meals. On this treatment she made a complete recovery. She would take the iodide potassium for two weeks and then leave it off for a like period. In this way she avoided iodism.

Mr. C. H. R., aged forty-one, had asthma for a year. He had an attack of influenza a year ago and since that time had had a mild bronchitis. During this time he had begun to have attacks of difficult breathing, and they had grown more frequent and frequently lasted an hour. I relied upon the nitroglycerin, strychnine and morphine tablets to relieve these attacks and was never disappointed in them. On cod-liver oil and iodide potassium given as in the first case he got along well, and steadily improved, and is now entirely well.

Mrs. L. F. Q., aged forty, had been a great sufferer for the past four years with asthma. The attacks exceeded in their violence and persistency any case I ever saw. The physician who had previously treated her relied upon the inhalation of chloroform to relieve the dyspnea, he having tried all the usual remedies to no purpose. When called first to this patient I lost no time in giving her a hypodermic injection of the nitroglycerin, strychnine and morphine tablets and found my patient got relief in a very short time. She subsequently used these tablets hypodermically when she felt the least premonition of an attack of asthma, and in this way aborted the attack. On these and iodide potassium, plus the regular administration of cod-liver oil she got along well, and she has now stopped the employment of the medicines and has now gone some time without the use of anything.

I can therefore most heartily recommend Sharp & Dohme's asthmatic hypodermics.

